# Intracranial neuromodulation for pediatric drug-resistant epilepsy: early institutional experience

**DOI:** 10.3389/fsurg.2025.1569360

**Published:** 2025-04-08

**Authors:** Julie Uchitel, Austin Lui, Juliet Knowles, Jonathon J. Parker, H. Westley Phillips, Casey H. Halpern, Gerald A. Grant, Vivek P. Buch, Ann Hyslop, Kevin K. Kumar

**Affiliations:** ^1^ Department of Neurosurgery, Stanford University School of Medicine, Stanford, CA, United States; ^2^ College of Osteopathic Medicine, Touro University California, Vallejo, CA, United States; ^3^ Department of Pediatric Neurology, Stanford University School of Medicine, Stanford, CA, United States; ^4^ Department of Neurology, Stanford University School of Medicine, Stanford, CA, United States; ^5^ Department of Neurosurgery, Mayo Clinic, Scottsdale, AZ, United States; ^6^ Division of Pediatric Neurosurgery, Lucile Packard Children’s Hospital, Palo Alto, CA, United States; ^7^ Department of Neurosurgery, Hospital of the University of Pennsylvania, Philadelphia, PA, United States; ^8^ Department of Neurosurgery, Duke University, Durham, NC, United States; ^9^ Department of Neurosurgery, Dell Medical School, The University of Texas at Austin, Austin, TX, United States

**Keywords:** epilepsy, DBS, RNS, pediatric epilepsy, VNS, neuromodulation

## Abstract

**Introduction:**

Pediatric drug-resistant epilepsy (DRE) is defined as epilepsy that is not controlled by two or more appropriately chosen and dosed anti-seizure medications (ASMs). When alternative therapies or surgical intervention is not viable or efficacious, advanced options like deep brain stimulation (DBS) or responsive neurostimulation (RNS) may be considered.

**Objective:**

Describe the Stanford early institutional experience with DBS and RNS in pediatric DRE patients.

**Methods:**

Retrospective chart review of seizure characteristics, prior therapies, neurosurgical operative reports, and postoperative outcome data in pediatric DRE patients who underwent DBS or RNS placement.

**Results:**

Nine patients had DBS at 16.0 ± 0.9 years and 8 had RNS at 15.3 ± 1.7 years (mean ± SE). DBS targets included the centromedian nucleus of the thalamus (78% of DBS patients), anterior nucleus of the thalamus (11%), and pulvinar (11%). RNS placement was guided by stereo-EEG and/or intracranial monitoring in all RNS patients (100%). RNS targets included specific seizure onset zones (63% of RNS patients), bilateral hippocampi (25%) and bilateral temporal lobes (12%). Only DBS patients had prior trials of ketogenic diet (56%) and VNS therapy (67%). Four DBS patients (44%) had prior neurosurgical interventions, including callosotomy (22%) and focal resection (11%). One RNS patient (13%) and one DBS patient (11%) required revision surgery. Two DBS patients (22%) developed postoperative complications. Three RNS patients (38%) underwent additional resections; one RNS patient had electrocorticography recordings for seizure mapping before surgery. For patients with a follow-up of at ≥1 year (*n* = 7 for DBS and *n* = 5 for RNS), all patients had reduced seizure burden. Clinical seizure freedom was achieved in 80% of RNS patients and 20% had a >90% reduction in seizure burden. The majority (71%) of DBS patients had a ≥50% reduction in seizures. No patients experienced no change or worsening of seizure frequency.

**Conclusion:**

In the early Stanford experience, DBS was used as a palliatively for generalized or mixed DRE refractory to other resective or modulatory approaches. RNS was used for multifocal DRE with a clear seizure focus on stereo-EEG and no prior surgical interventions. Both modalities reduced seizure burden across all patients. RNS offers the additional benefit of providing data to guide future surgical planning.

## Introduction

Epilepsy is one of the most common neurological disorders in children, affecting one in every 150 children before ten years of age (1). Drug-resistant epilepsy (DRE), also referred to as refractory epilepsy, accounts for approximately one-third of all epilepsy cases (2). The International League Against Epilepsy (ILAE) defines DRE as the persistence of seizures despite trials of two appropriate anti-seizure medications (ASMs), either as monotherapy or in combination (3). For patients with DRE, invasive neurosurgical interventions may include resection, laser ablation, or disconnection procedures (4, 5). When these conventional surgical approaches are not appropriate or effective, neurostimulation therapies such as vagal nerve stimulation (VNS), deep brain stimulation (DBS), and responsive neurostimulation (RNS) may be considered as alternative treatment options (6).

VNS has been a well-established treatment for pediatric focal epilepsy for over two decades (7, 8). VNS modulates seizure activity through the left vagus nerve, which projects to brainstem nuclei that give rise to diffuse sub-cortical and cortical connections (9). Early clinical trials demonstrated that approximately 30% of patients achieve greater than 50% reduction in seizure frequency in the first year of use (10). Initially approved by the FDA in 1997 for patients aged 12 and older (11), VNS received expanded approval in 2005 for children as young as 4 years old (12). More recent studies have detailed its safety, efficacy in reducing seizure burden, and improvements in quality of life (7, 8, 13–16). Due to its extracranial nature, VNS may be favored for DRE before more invasive intracranial neuromodulation methods are pursued.

DBS and RNS are more recently developed neuromodulation techniques. DBS delivers electrical stimulation via implanted intracranial electrodes targeting thalamocortical connections involved in seizure generation (17). The most common DBS targets are the anterior nucleus of the thalamus (ANT) or the centromedian nucleus (CMN) (6, 18), both of which play roles in modulating seizure propagation and cortical excitability (19–21). A DBS external pulse generator implanted in the chest provides stimulation through stimulation parameters that are programmed and adjusted in the clinic. RNS, in contrast, senses and responds to abnormal local field potentials in real time, providing targeted electrical stimulation to prevent seizure activity. Placement of RNS electrodes in pediatric patients most commonly targets the frontal lobe, mesial temporal structures, and the thalamus (22).

Following the landmark Stimulation of the Anterior Nucleus of the Thalamus for Epilepsy (SANTE) trial, DBS received FDA approval in 2018 for the treatment of drug resistant focal epilepsy in adults (6, 18). Similarly, RNS received FDA approval for the treatment of focal epilepsy in adults in 2013 (23, 24). Given their more recent development, DBS and RNS have been less well evaluated as compared to VNS, especially in pediatric patients. For pediatric patients, DBS and RNS are used off-label, which has hindered the process of achieving FDA approval (25).

Nonetheless, DBS and RNS have shown promising outcomes in reducing seizure burden across several studies. In a systematic review of DBS and RNS in pediatric patients, 75% of pediatric patients treated with DBS (*n* = 72) experienced >50% seizure reduction, with 6 achieving seizure freedom, after a median follow-up (FU) of 14 months (19). In the pediatric patients treated with RNS (*n* = 46), 73% had >50% seizure reduction at a median follow up of 22 months, including 4 patients who achieved seizure freedom (19). Similarly, a meta-analysis of 40 pediatric DRE patients treated with DBS reported that 85% had seizure reduction, with 13% achieving seizure freedom (26). While DBS and RNS are generally reported as safe for children, reported complications include worsening of seizures (19, 27), increased behavioral aggression (27), need for hardware revision (27, 28), infections (15, 26, 28), skin erosions (26), and hardware failure (26, 29).

In this study, we describe the early institutional experience of Stanford with DBS and RNS in pediatric patients with DRE. This early series contributes to emerging indications for pediatric neuromodulation, offering insight into its unique challenges in patient selection, timing, and intracranial target. Our goal is to further refine the understanding of pediatric neuromodulation and contribute to the growing body of evidence that will inform clinical decision-making and future research in this field.

## Methods

We identified 17 pediatric DRE patients who were considered for intracranial neuromodulation (DBS and RNS) at Stanford Lucile Packard Children's Hospital between 2013 and 2024. These patients were identified from a comprehensive list of all patients who underwent pediatric neuromodulation for epilepsy at Stanford. A small subset of RNS patients were previously reported in a multicenter study (30). Basic demographic information, seizure history, prior therapies trialed, neurosurgical operative reports, and postoperative seizure and outcome data were analyzed from each patient's medical records.

### Patient selection for neuromodulation

Each patient in our center underwent extensive evaluation by our pediatric neurology and neurosurgery teams before pursuing neurosurgical intervention. This includes discussion at our weekly Epilepsy Surgery Conference, consisting of a panel of neurosurgeons, epileptologists, neuroradiologists, neurologists, advanced practice providers, and neuropsychologists. Evaluation included assessment of each patient's MRI, video EEG (vEEG), functional MRI, diagnostic intracranial EEG with either cortical grids/strips or stereo-EEG (sEEG), magnetoencephalography (MEG), and positron emission tomography (PET), as indicated.

### Study variables

Seizure semiology was determined using a combination of patients' clinical features and seizure localization on inpatient vEEG. DRE was defined as failure to control seizures using adequate trials of at least two ASMs (3). Data collected on implantation technique included prior sEEG or cortical grid/strip monitoring, device implanted, and subsequent neurosurgical interventions, among other variables. For response to neuromodulation, each patient was placed into 1 of 5 categorical outcomes: (1) free of disabling seizures; (2) ≥90% reduction of disabling seizures; (3) ≥50% but <90% reduction of disabling seizures; (4) <50% but some noticeable reduction of disabling seizures; or (5) no improvement (30–33). Outcomes were only considered for patients who had a follow up of at least 1 year or more to allow for sufficient time for neuromodulation to have an effect. Post-surgical complications, revisions, explants, and device side effects were also noted.

## Results

### Patient demographics and clinical characteristics

A total of 17 patients underwent device implantation, 9 with DBS and 8 with RNS. The demographic and clinical characteristics are summarized in Table 1. The age at epilepsy onset was 4.2 ± 1.3 years for DBS patients (mean ± SE). For RNS patients, age at epilepsy onset was 8.0 ± 1.5 years. The seizure semiology differed between groups, with generalized seizures being more common in DBS patients (78%) compared to RNS patients (12%). DBS patients more frequently presented with more than one seizure type as compared to RNS patients and had greater frequency of daily or weekly seizures. Details on seizure burden and frequency for both groups are detailed in Table 2.

**Table 1:** Cohort demographics.

Variable	Total Count (%) or mean ± SE
Patients	17 (100%)
Male	9 (53%)
Female	8 (47%)
Race	
Asian	1 (6%)
Other	1 (6%)
White	10 (59%)
Unknown	5 (29%)
Ethnicity	
Hispanic or Latino	5 (29%)
Not Hispanic or Latino	10 (59%)
Unknown	2 (12%)
Age at epilepsy onset (years)	6.0 ± 1.1
Semiology	
Focal	3 (18%)
Multifocal	6 (35%)
Generalized	8 (47%)
Neuromodulation device	
DBS	9 (53%)
RNS	8 (47%)
Pre-DBS/RNS seizure frequency	
Daily	9 (53%)
Weekly	4 (25%)
Monthly	1 (7%)
>Monthly	5 (36%)

**Table 2:** Overview of DBS and RNS in pediatric patients with DRE.

Variable	DBS Count (%) or mean ± SE	RNS Count (%) or mean ± SE
Number of patients	9 (100%)	8 (100%)
Male	4 (44%)	5 (62%)
Age epilepsy onset (years)	4.2 ± 1.3	8.0 ± 1.5
Seizure semiology		
Focal	1 (11%)	2 (25%)
Multifocal	1 (11%)	5 (63%)
Generalized	7 (78%)	1 (12%)
Prior failed interventions		
ASMs	9 (100%)	8 (100%)
Ketogenic diet	5 (56%)	0 (0%)
VNS	6 (67%)	0 (0%)
Resection	1 (11%)	0 (0%)
Callosotomy	2 (22%)	0 (0%)
Other	1 (11%)	0 (0%)
# ASMs at time of evaluation	3.7 ± 0.5	2.8 ± 0.3
sEEG monitoring	1 (11%)	8 (100%)
Intracranial grids/strip monitoring	1 (11%)	2 (25%)
Age at DBS/RNS placement (years)	16.0 ± 0.9	15.3 ± 1.7
Headframe Used		
Mayfield	1 (14%)	7 (88%)
Leksell	5 (71%)	1 (12%)
Frameless	1 (14%)	0 (0%)
Intraoperative CT or MRI	9 (100%)	6 (75%)
Device implanted		
Boston scientific (DBS)	8 (89%)	–
Medtronic (DBS)	1 (11%)	–
NeuroPace (RNS)	–	8 (100%)
Brain region targeted		
CMN	7 (78%)	0 (0%)
ANT	1 (11%)	0 (0%)
Pulvinar nucleus	1 (11%)	0 (0%)
Bilateral hippocampi	0 (0%)	2 (25%)
Bilateral temporal lobes	0 (0%)	1 (12%)
Seizure onset focus or foci	0 (0%)	5 (63%)
Postoperative complications	2 (22%)	0 (0%)
Infection	1 (11%)	0 (0%)
Intracranial hemorrhage	0 (0%)	0 (0%)
Stroke	0 (0%)	0 (0%)
Device malfunction or migration	0 (0%)	0 (0%)
Other	1 (11%)	0 (0%)
Additional post-neuromodulation surgeries		
Resection	0 (0%)	3 (38%)
Thermal ablation	0 (0%)	0 (0%)
Other	0 (0%)	1 (12%)
Revision required	1 (11%)	2 (25%)
Time after implant (years)	0.9 ± 0	1.6 ± 0
Battery revision	1 (11%)	0 (0%)
Lead replacement	0 (0%)	1 (12%)
Explant required	1 (11%)	0 (0%)

ASM, antiseizure medication; sEEG, stereo-electroencephalography; VNS, vagus nerve stimulator; DBS, deep brain stimulation; RNS, responsive neurostimulation; CMT, centromedian nucleus of the thalamus; ANT, anterior nucleus of the thalamus.

### Failed prior therapies and surgical interventions

All patients had failed at least two ASMs and were classified as having DRE. The mean number of ASMs at time of presurgical evaluation was similar between the groups (DBS: 3.7 ± 0.5; RNS: 2.8 ± 0.3). DBS patients had prior trials of ketogenic diet (56%) and VNS therapy (67%). Patients who had prior VNS therapy had VNS for 4.8 ± 0.6 years (range 2.4–7.3) with stimulation parameter adjustment before undergoing DBS placement. No patients trialed these therapies in the RNS cohort.

Four DBS patients (44%) had prior neurosurgical interventions aimed at controlling seizures, while no RNS patients had prior neurosurgical interventions. Among the four DBS patients, two patients underwent callosotomy, one patient underwent focal resection, and one patient underwent placement and revision of a ventriculoperitoneal shunt. Patient 2 with Lennox-Gastaut Syndrome (LGS) underwent a 2/3 corpus callosotomy which was partially helpful in decreasing seizure burden; however, she continued to have seizures after surgery and had resultant left-sided weakness and supplementary motor area syndrome. Patient 4, also with LGS, first underwent an anterior 2/3 callosotomy, and then later underwent an additional craniotomy for ablation of the splenium of the corpus callosum. Patient 6 with underwent a right anterior temporal lobectomy as well as right frontopolar resection.

### Device selection and rationale

Device selection and surgical targeting were tailored to the patients' specific clinical profiles, including epilepsy etiology, seizure semiology, trialed therapies (ASMs, ketogenic diet, VNS), and prior neurosurgical interventions (Table 3). DBS was more often used for used for generalized epilepsy, particularly in patients with LGS or other generalized epilepsy syndromes (78%), targeting the centromedian nucleus (CMN) in most cases (78%). In contrast, RNS was employed mostly in patients with focal or multifocal epilepsy (88%), guided by invasive monitoring such as sEEG or grid/strip intracranial recordings. The rationale for device selection often reflected a balance between seizure control and preserving functional cortex, with RNS prioritized when seizure foci overlapped eloquent cortex (e.g., motor or somatosensory regions, see Patients 11 and 13).

**Table 3:** Rationale for RNS vs. DBS by patient.

Patient	Epilepsy type	Age (years)	RNS or DBS	Target	Rationale
1	LGS	16	DBS	CMN	DRE. VNS with limited efficacy. Prior SE and ALL in remission. Behavioral issues. vEEG showed diffuse generalized abnormalities
2	LGS due to *NEXMIF* mutation	19	DBS	CMN	DRE. VNS not effective. Side effects from ASMs. Prior anterior 2/3 callosotomy with minor improvement. Post-surgical left-sided weakness and supplemental motor area syndrome. Behavioral issues. vEEG showed left hemispheric spikes, generalized fast activity/polyspikes, diffuse slowing
3	Generalized epilepsy, idiopathic (VUS in *SETD1B)*	16	DBS	CMN	DRE. Prior treatment with VNS limited efficacy. vEEG showed generalized spikes, bifrontally predominant
4	LGS	15	DBS	CMN	DRE. VNS limited efficacy. Previous callosotomy with some relief of seizures. vEEG showed GPFA and 3–4 Hz generalized spike and wave discharges
5	Generalized idiopathic epilepsy	13	DBS	CMN	DRE VNS not effective. Parents concerned about seizures as safety risk and note clear negative effects on cognition. vEEG showed mild diffuse background slowing
6	Focal idiopathic epilepsy	21	DBS	ANT	DRE. Persistent seizures after previous right anterior temporal lobectomy and right frontopolar resection. vEEG after resection showed interictal right central sharps and SE
7	LGS	12	DBS	CMN	DRE. Prior ketogenic diet. Side effects from medications. vEEG showed abundant multifocal sharp and spike and wave discharges. sEEG monitoring considered but not done due to procedure-related risks outweighing possibility of identifying seizure focus
8	LGS	17	DBS	CMN	DRE. VNS limited efficacy. Multifocal onset epilepsy. sEEG demonstrated largest amplitudes in the frontotemporal region but did not identify seizure onset zone.
9	Hydrocephalus, left hemiparesis, and bilateral epilepsy due venous hemorrhage secondary to perinatal venous sinus thrombosis	16	DBS	GPi, right CMN, left pulvinar	DRE and severe dystonia. VP shunt in place. DBS implant targeting GPi for treatment of dystonia and right CMN for right-sided seizures. Left pulvinar stimulation not started at time of publication due to no left-sided seizure occurrence in recent years. vEEG showed right hemispheric slowing and multifocal spiking
10	Bilateral temporal epilepsy, idiopathic	19	RNS	Bilateral hippocampi	DRE. sEEG showed bilateral onset of seizures in the distal left mesial temporal, distal left posterior temporal, and right lateral frontal regions
11	Focal epilepsy secondary to left parietal focal cortical dysplasia, type 1A	20	RNS	Left anterior parietal cortex	DRE. Intracranial grid recording and sEEG recordings demonstrated seizure onset in primary somatosensory cortex. Concern for permanent motor deficit with resection
12	Bilateral temporal lobe epilepsy, idiopathic	19	RNS	Bilateral hippocampi	DRE. sEEG demonstrated bitemporal onset of the hippocampi. Concern for memory deficit with dominant mesial temporal ablation
13	Focal epilepsy, idiopathic	12	RNS	Right motor cortex	DRE. Intracranial grid/strip recording revealed seizure onset zone in right motor cortex. Concern for permanent motor deficit with resection
14	Left temporal and parietal epilepsy, idiopathic	15	RNS	Canthus, middle temporal gyrus	DRE. sEEG demonstrating seizure onset zone over left temporal and parietal regions
15	Bilateral temporal epilepsy, idiopathic	18	RNS	Bilateral temporal regions	DRE. sEEG showed bilateral temporal epileptogenicity
16	Right-sided epilepsy, DEE-SWAS	7	RNS	Right insula and right frontal operculum	DRE and DEE-SWAS. sEEG revealed epileptogenicity in right insula and right frontal operculum responsive to low frequency stimulation on ESM
17	Bilateral occipital lobe epilepsy secondary to neonatal hypoglycemia and resultant encephalomalacia	11	RNS	Bilateral occipital lobes	DRE. sEEG demonstrate independent seizure onset zones in bilateral occipital lobes

DBS, deep brain stimulation; RNS, responsive neurostimulation; LGS, Lennox-Gastaut syndrome; DRE, drug resistant epilepsy; SE, status epilepticus; CMN, centromedian nucleus of the thalamus; ANT, anterior nucleus of the thalamus; GPFA, generalized paroxysmal fast activity; GPi, globus pallidus interna; sEEG, stereo-electroencephalography; VUS, variant of unknown significance; vEEG, video EEG; ASM, antiseizure medication; DEE-SWAS, developmental epileptic encephalopathy with spike waves activation in sleep; ESM, electrical stimulation mapping.

### Perioperative data

All surgeries were performed under general anesthesia. sEEG monitoring with ROSA was utilized in 11% of DBS patients compared to 100% of RNS patients (Table 2). The mean age at the time of neuromodulation device placement was 16.8 ± 0.9 years for DBS patients and 15.3 ± 1.7 years for RNS patients.

**Figure 1:**
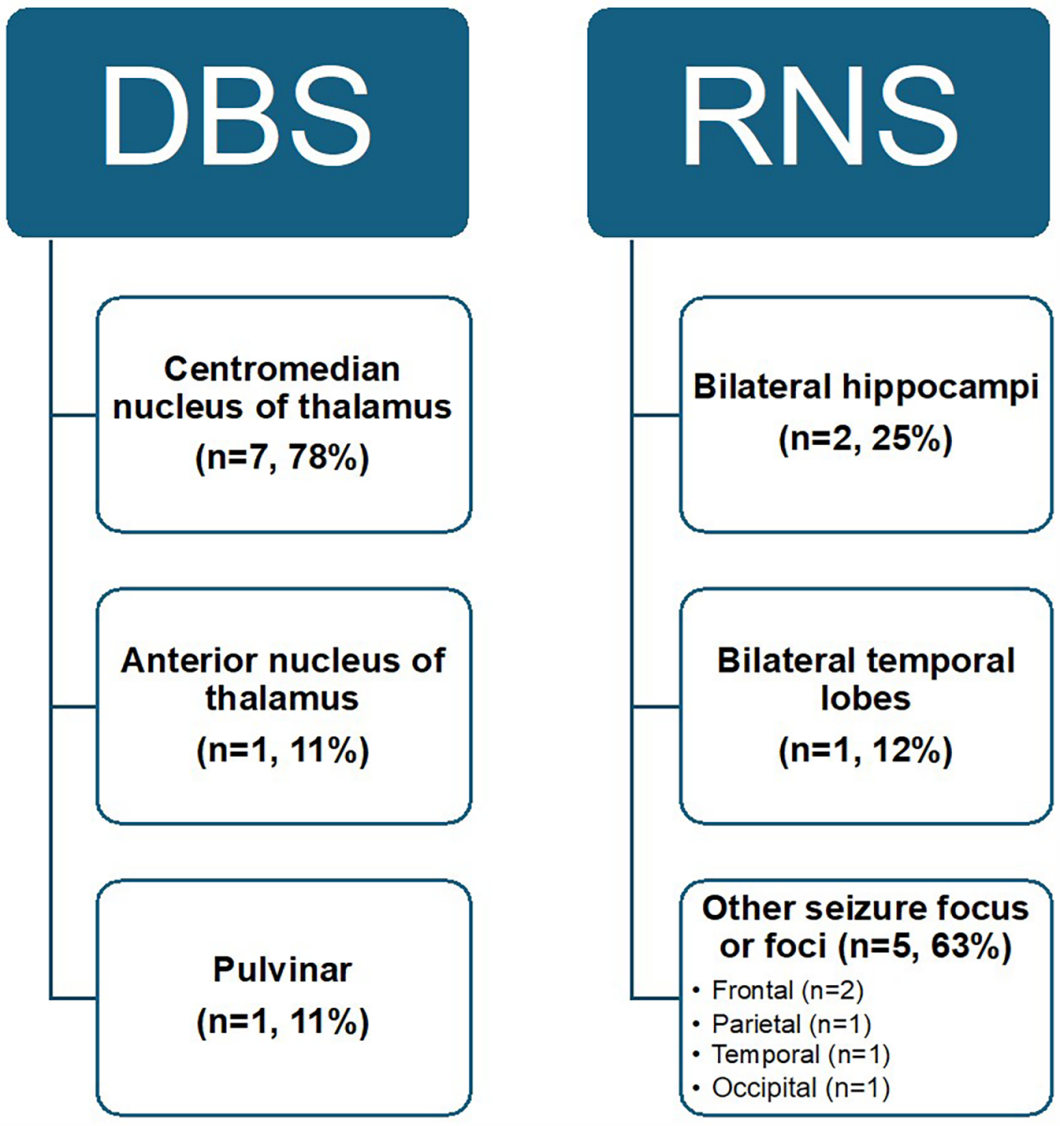
Overview of neuromodulation targets in patients with pediatric drug-resistant epilepsy. Targets for DBS include the centromedian nucleus of the thalamus (CMN), anterior nucleus (ANT), and pulvinar. Targets for RNS include the bilateral hippocampi, bilateral temporal lobes, or another seizure focus or foci identified on sEEG. These targets specifically are in the right insula and right frontal operculum, right motor cortex, left anterior parietal cortex, canthus and middle temporal gyrus, and the bilateral temporal and occipital lobes.

**Figure 2:**
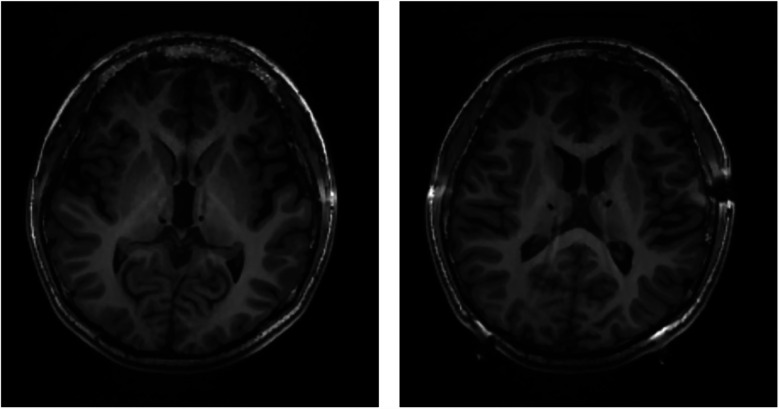
DBS in the CMN of the thalamus in a pediatric patient with drug-resistant epilepsy. Postoperative MRI scans from Patient 1 for visualization of DBS lead placement. DBS leads can be seen as black circles targeting the centromedian nucleus of the thalamus (CMN). The left image is dorsal to the right image.

The most common DBS target location was the CMN (78%), followed by the ANT (11%), and the pulvinar nucleus of the thalamus (11%). All RNS patients underwent sEEG recording which was used to determine placement location, most often a seizure onset focus or foci (63%), followed by the bilateral hippocampi (25%) or bilateral temporal lobes (12%) (Figures 1, 2). Identified seizure onset foci were the in the left anterior parietal region, the right motor cortex, the canthus/middle temporal gyrus, and the right insula and right frontal operculum.

### Postoperative complications

Postoperative complications occurred two DBS patients. Patient 1 experienced a prolonged 14-day postoperative recovery, characterized by waxing and waning mental status, respiratory distress requiring BIPAP, prolonged fevers, and labile blood pressures and fluid status (Table 3). Contributing factors likely included stress from pneumonia, underlying poor reserves, ICU delirium, and hyperammonemia encephalopathy, with seizures managed through adjustments in ASMs. Patient 4 developed swelling and redness along her DBS site and chest incision after implant of the pulse generator. This later became infected, requiring readmission, return to the operating room, surgical exploration, and explant 6 weeks after initial implant. The patient was treated with 2 weeks of IV antibiotics. No RNS patients had postoperative complications.

Revision surgery was necessary in one patient with RNS (13%) and one with DBS (11%). Patient 14 underwent a repositioning and replacement of RNS leads in conjunction with a resection of the left temporal pole and amygdala 1.6 years after initial placement. Patient 1 had their DBS pulse generator implanted deep to the pectoral muscle initially which led to difficulty charging the device. The patient underwent revision surgery 11 months after initial surgery to have the pulse generator placed superficial to the muscle, which resolved the issue with charging.

### Additional surgical interventions after neuromodulation

Three RNS patients underwent additional neurosurgical interventions during or following RNS placement. Patient 11 underwent resection of a left parietal epileptic focus during the same surgery as RNS placement. Patient 13 underwent three resective surgeries after RNS placement: the first consisted of resections around the motor and sensory areas, the second was resection of a right frontal epileptic focus, and the third involved a resection near the motor strip, which resulted in an infected subgaleal fluid collection requiring drainage.

In Patient 14, post-implantation RNS was actively used to map seizures and guide decisions regarding future resections. Despite ongoing adjustments to RNS settings, the patient continued to experience daily seizures. Over multiple clinic visits, RNS data consistently localized seizure onset to the left mesial temporal lobe, specifically the hippocampus, where the hippocampal lead showed significant ictal activity compared to a lateral strip electrode. This information guided the decision to consider hippocampal transection as a memory-preserving surgical option given the patient's high verbal memory function. This was favored over laser ablation, which was more likely to negatively impact memory. After this transection, the patient continued to have cluster seizures prompting a second craniotomy for left amygdalectomy and temporal pole resection with RNS lead repositioning. After this repositioning of RNS leads, the RNS system continued to capture seizures, supporting that the epileptic tissue had not been fully resected. The patient underwent a third resection surgery consisting of a temporal lobectomy and replacement of RNS leads. As of the time of publication, three years after initial RNS placement, the patient has achieved clinical seizure freedom.

### Side effects

Only one DBS patient had a side effect of worsened symptoms of depression noted two years after initial implant. This resolved once the patient began seeing a counselor. No RNS patients had documented side effects.

### Long term outcomes

Among the 12 patients with a follow up of one year or more (7 DBS, 5 RNS), the mean age at last follow up was 20.5 ± 1.3 years for DBS patients and 22.1 ± 1.4 years for RNS patients. The mean duration of follow-up was 4.0 ± 0.4 years for DBS patients and 4.8 ± 0.8 years for RNS patients. At this time, the mean number of ASMs was 3.9 ± 0.6 for DBS patients and 2.0 ± 0.4 for RNS patients. ASM burden decreased in 14% of DBS patients and 50% of RNS patients.

All patients had reduced seizure burden relative to pre-neuromodulation. Of the RNS patients with at least a 1 year follow up, 100% (*n* = 5) had a >90% reduction in seizure burden, and 80% achieved a seizure freedom. Of the DBS patients with at least a 1 year follow up, 71% of DBS patients had a ≥50% reduction in seizures and 28% had a <50% reduction. Among the DBS patients with CMN as a target (*n* = 6), 67% percent had a ≥50% reduction in seizure burden and 33% had a <50% reduction. No patients experienced a lack of effect or worsening of seizures with either device-based therapy. The change in seizure burden after therapy is presented in Table 4.

**Table 4:** Outcomes after RNS and DBS (as documented at last FU).

Variable	DBS, patients with ≥1 year FU Count (%) or mean ± SE	RNS, patients with ≥1 year FU Count (%) or mean ± SE
Number	7 (100%)	5 (100%)
Pre-DBS/RNS number of Seizure types	1.9 ± 0.3 (range: 1–3)	1.4 ± 0.2 (range: 1–2)
GTC seizures	7 (100%)	2 (40%)
Pre-DBS/RNS seizure frequency		
Daily	5 (56%)	2 (40%)
Weekly	2 (22%)	1 (20%)
Monthly	0 (0%)	0 (0%)
>Monthly	0 (0%)	2 (40%)
Age at last FU (years)	20.5 ± 1.3	22.1 ± 1.4
Duration of FU (years)	4.0 ± 0.4	4.8 ± 0.8
Number of seizure types (in those not seizure-free)	1.0 ± 0	0 ± 0.2 (range: 0–1)
GTC seizures	4 (57%)	0 (0%)
Seizure frequency		
Daily	5 (71%)	0 (0%)
Weekly	1 (14%)	0 (0%)
Monthly	0 (0%)	0 (0%)
>Monthly	1 (14%)	1 (20%)
No seizures	0 (0%)	4 (80%)
Number of ASMs	3.9 ± 0.6	2.0 ± 0.4
Increased # from pre-surgery	2 (28%)	1 (12%)
Same # from pre-surgery	4 (57%)	3 (38%)
Decreased # from pre-surgery	1 (14%)	4 (50%)
Side effects	1 (14%)*	0 (0%)
Change in seizure burden		
Seizure free	0 (0%)	4 (80%)
>90% reduction	1 (14%)	1 (20%)
≥50% reduction	4 (57%)	0 (0%)
<50% reduction	2 (28%)	0 (0%)
No change	0 (0%)	0 (0%)
Worse	0 (0%)	0 (0%)

^a^ One patient with DBS had worsening of previously diagnosed depression that resolved with psychological counseling. FU, follow up; DBS, deep brain stimulation; RNS, responsive neurostimulation; GTC, generalized tonic-clonic.

## Discussion

This study presents the early institutional experience with DBS and RNS in a cohort of 17 pediatric patients with DRE. DBS was typically used as a palliative procedure for patients with generalized epilepsy that was highly refractory to prior resection and modulatory approaches. RNS was mostly used for multifocal or focal epilepsy patients with a clear sEEG focus of onset and no prior neurosurgical interventions. Both DBS and RNS were relatively safe, with postoperative complications occurring in two DBS patients, and revision surgery required in one RNS patient. RNS also proved useful in guiding subsequent resection surgeries for one patient. Both modalities demonstrated improvements in seizure outcomes, with 100% of RNS patients and 71% of DBS patients achieving a ≥50% reduction in seizure burden at last follow up of at least one year duration.

### Choice of neuromodulation vs. resection or ablation

A multifactorial approach that considers a patient's prior epilepsy history, neuroimaging findings, genetic testing, and ASM response is necessary to decide between neuromodulation vs. resection or ablation. Resection surgery is the first choice when a focal lesion is clearly identified. Larger resections or hemispherectomy may be necessary when multifocal or unilateral diffuse lesions are present, although this poses challenges when eloquent cortex is present (e.g., bitemporal, primary motor/sensory, or language areas) (22, 34). Neuromodulation may be a better option in patients who have complex, bilateral epileptogenic networks, such as those with LGS (35). In patients with LGS, DBS has been shown to reduce seizure burden and also improve attention, behavioral issues, and quality of life (36, 37). Lesions in eloquent cortex are also an indication for possible neuromodulation. Patients with average or above average language and memory function may experience significant postoperative impairments if regions such as the hippocampus are resected or ablated. This was the case for several patients who received RNS in our study. Finally, neuromodulation can serve as a palliative option when prior drug therapies and surgical interventions resection or have failed, as was the case for 44% of DBS patients in our study.

### DBS indications and outcomes

In this cohort, DBS was typically used as a palliative procedure for patients with generalized epilepsy (78%) who had undergone multiple prior neurosurgical interventions (44%) and were also refractory to VNS (67%) and ketogenic diet therapy (56%). In addition, DBS patients who received VNS had a mean therapy duration of 4.8 ± 0.6 years (range 2.4–7.3) before DBS. Of note, the DBS patients in this cohort appeared had more severe epilepsy than the RNS patients, with 100% of DBS patients presenting with GTC seizures vs. 40% of RNS patients, an earlier mean age of seizure onset in DBS patients (4.2 vs. 8.0 years), and more DBS patients resistant to prior neurosurgical, dietary, and VNS interventions. Prior work, including a partially randomized trial, has shown that DBS as an add-on to VNS therapy is more beneficial than VNS with continued parameter tuning alone (36, 38, 39). The potential synergistic effects of DBS and VNS are not well understood and may involve the modulation of multiple networks (38).

Of the DBS patients who had a least a one-year follow up, 71% had a ≥50% reduction in seizures as of last follow up. This is similar to that of a large review by Khan et al., where 75% of patients had at least a ≥50% seizure reduction (19). Most patients in our study had electrodes targeting the CMN, and 67% of these patients experienced a ≥50% reduction in seizure burden. The CMN was shown to be a promising target in the prospective, double-blind randomized ESTEL trial (40). Other potential targets, such as the ANT, may also be further considered, as it is currently approved for treating focal epilepsy in adults (6, 18). This was the target of one DBS patient in our cohort who demonstrated a >90% seizure reduction at last FU.

### RNS indications and outcomes

RNS is particularly useful for patients with focal or multifocal epilepsy where the stimulating electrode can be placed into or on top of the cortical seizure onset zone, even when it overlaps with eloquent cortex. For our RNS patients, the majority had focal or multifocal epilepsy (88%) with an older age of seizure onset (mean 8.0 ± 4.2 years), and none had prior failed neurosurgical interventions. As performed for all RNS patients in our study, sEEG and grid/strip intracranial monitoring can provide precise seizure localization to optimize placement of the RNS electrode. However, modulation of the seizure onset zone may not be sufficient for patients that have more diffuse epileptogenic networks, as is seen in many of those with extratemporal lobe epilepsy (41). More research is needed on how to identify whether a focus-driven or network-driven approach will be more effective in a given patient.

Among our RNS patients who had at least 1 year follow up, 80% achieved seizure freedom and 100% achieved a ≥ 50% reduction in seizure frequency as of last follow up. This data is consistent with prior reports of RNS in pediatric DRE. Swartwood et al. reported a cohort of 22 patients, 64% of whom demonstrated a ≥50% reduction in seizure frequency at a mean follow up of 19 months (31). Similarly, in a multi-center study by Nagahama et al., 59% of 17 pediatric DRE patients achieved a ≥50% seizure reduction (30).

#### RNS to interrogate possible surgical resection zones

In addition to its therapeutic effect, RNS electrocorticography recordings may confirm localization of the epileptogenic zone, helping guide future resective surgery. One of our patients underwent three resective surgeries with improved precision using RNS-recorded seizures to identify the target area. In this respect, RNS can act as an out-of-hospital monitoring system, allowing for both disruption of seizure activity as it occurs and recording of activity to guide future treatment. Additionally, RNS may be useful in mapping diffuse epileptic networks with multiple contributing nodes (42). Implanting leads across these nodes can help identify their activity levels, enabling targeted resection most likely to improve outcomes. Additional work is needed on how to best use RNS-captured seizures to guide subsequent surgical intervention.

### Future outlook: improved targeted patient selection

Future research should focus on identifying patient selection criteria for RNS and DBS, optimization of the timeliness of device placement, evaluating long-term seizure and quality-of-life outcomes, and developing innovative strategies to map and treat diffuse epileptic networks. Current clinical decision-making often prioritizes identifying candidates for focal resection or callosotomy based on seizure type, with neuromodulation considered as a subsequent or palliative procedure only after other interventions fail. However, there is a need to shift toward a more strategic, biomarker-driven approach that identifies patients who are not ideal resective surgical candidates from the outset. Understanding when a patient's epileptic network is too large or complex for resection could allow for earlier selection for neuromodulation as the primary intervention, sparing patients a series of invasive and ineffective procedures. The impact of neuromodulation on quality of life as compared to other approaches warrants further consideration. Moreover, future studies should focus on defining the clinical and neurophysiological markers that can guide preoperative decision making.

Recent research efforts have highlighted the potential of individual differences in the functional and structural connectivity of thalamocortical networks as biomarkers for optimizing patient selection, stimulation parameters, and neuromodulation targets (43–45). Local field potentials, which may be recorded with newer DBS devices, may also be helpful in optimizing programming settings or providing closed-loop stimulation (46, 47). Integrating machine learning and artificial intelligence into neuromodulation devices may enable dynamic seizure prediction, or aid in the optimization of individualized stimulation parameters in the future. Currently underutilized or unidentified clinical or neuroimaging biomarkers may prove important, indicating a need for further research associated with patient selection and improving outcomes.

Limitations of this study include the cohort size, the potential selection bias of these patients, that this is a single-institution study, and that some patients underwent other epilepsy surgeries that may have had a contributory effect to the results presented here. Nonetheless, our study demonstrates the safety and efficacy of DBS and RNS in a relatively clinically diverse cohort of patients with long-term follow up in most patients.

## Conclusion

This study presents our early institutional experience of DBS and RNS in pediatric DRE. Both techniques demonstrated improvement in reducing seizure burden, with 100% of RNS patients with predominantly multifocal epilepsy and 71% of DBS patients with primarily generalized epilepsy having a ≥50% reduction in seizures. Post-surgical complications occurred in two DBS patients and one RNS patient. RNS offers unique utility in capturing long-term seizure-related data, supporting both immediate seizure control and long-term surgical planning. This study also highlights the efficacy of neuromodulation in patients following prior unsuccessful neurosurgical interventions and emphasizes the need to identify patients best suited for neuromodulation before irreversible resective surgery is utilized. Future efforts are needed to optimize patient selection criteria that integrates individual-specific clinical and neuroimaging biomarkers of epilepsy networks to guide intervention and timing.

## Data Availability

The original contributions presented in the study are included in the article/Supplementary Material, further inquiries can be directed to the corresponding authors.
